# Plasma Eicosanoid Profile in *Plasmodium vivax* Malaria: Clinical Analysis and Impacts of Self-Medication

**DOI:** 10.3389/fimmu.2019.02141

**Published:** 2019-09-18

**Authors:** Péricles Gama Abreu-Filho, Andrea Monteiro Tarragô, Allyson Guimarães Costa, Wuelton Marcelo Monteiro, Alyne Fávero Galvão Meielles, Thainá Cristina Cardoso Costa, Jéssica Santos Silva, Fabiana Albani Zambuzi, Luiz Gustavo Gardinassi, Luiz Alberto Beraldo Moraes, Marcus Vinícius Guimarães Lacerda, Carlos Arterio Sorgi, Lúcia Helena Faccioli, Adriana Malheiro

**Affiliations:** ^1^Programa de Pós-Graduação em Imunologia Básica e Aplicada, Instituto de Ciências Biológicas, Universidade Federal do Amazonas (UFAM), Manaus, Brazil; ^2^Programa de Pós-Graduação em Biociência e Biotecnologia, Faculdade de Ciências Farmacêuticas de Ribeirão Preto, Universidade de São Paulo (USP), Ribeirão Preto, Brazil; ^3^Departamento de Análises Clínicas, Toxicológicas e Bromatológicas, Faculdade de Ciências Farmacêuticas de Ribeirão Preto, Universidade de São Paulo (USP), Ribeirão Preto, Brazil; ^4^Departamento de Ensino e Pesquisa, Fundação Hospitalar de Hematologia e Hemoterapia do Amazonas (HEMOAM), Manaus, Brazil; ^5^Programa de Pós-Graduação em Medicina Tropical, Universidade do Estado do Amazonas (UEA), Manaus, Brazil; ^6^Instituto de Pesquisa Clínica Carlos Borborema, Fundação de Medicina Tropical Doutor Heitor Vieira Dourado (FMT-HVD), Manaus, Brazil; ^7^Programa de Pós-Graduação em Ciências Aplicadas à Hematologia, Universidade do Estado do Amazonas (UEA), Manaus, Brazil; ^8^Programa de Pós-Graduação da Bioquímica, Faculdade de Medicina de Ribeirão Preto, Universidade de São Paulo (USP), Ribeirão Preto, Brazil; ^9^Departamento de Química, Faculdade de Filosofia, Ciências e Letras de Ribeirão Preto, Universidade de São Paulo (USP), Ribeirão Preto, Brazil; ^10^Instituto de Pesquisas Leônidas & Maria Deane, FIOCRUZ-Amazônia, Manaus, Brazil

**Keywords:** eicosanoids, *Plasmodium vivax*, inflammatory mediators, cytokines, self-medication

## Abstract

The participation of cytokines and chemokines in *Plasmodium vivax* malaria (*Pv*-malaria) activates the immune response and thus causes the production of several inflammatory mediators. This process is already well-established, but little is known about eicosanoids in malaria physiopathology, especially in regards to inflammation and immunity. Malaria is an acute febrile syndrome similar to any other less important infectious disease and people may self-medicate with any anti-inflammatory drugs in order to cease the recurrent symptoms of the disease. Based on this information, the study describes the eicosanoid profile and its possible influence on the production of cytokines and chemokines in *P. vivax* infections. In addition, we investigated the influence of self-medication with anti-inflammatory drugs in this immune profile. Twenty-three patients were included in the study, with or without self-medication by anti-inflammatory drugs prior to diagnosis. A total 12 individuals were selected for the control group. Eicosanoid profiles were quantified by HPLC-MS/MS, and cytokines and chemokines by flow cytometry and ELISA. The *Pv*-malaria infection significantly reduces the production of several lipid mediators, and its action is increased by self-medication. We observed that the eicosanoids we found derive from the lipoxygenase and cyclooxygenase pathways, and present positive and negative correlations with chemokines and cytokines in the follow-up of patients. Our data suggest that self-medication may interfere in the immunological characteristics in *P. vivax* infection and may modify the follow-up of the disease.

## Introduction

Malaria is one of the oldest infectious diseases on earth, and often leads to severe disease or even death (1). Caused by the Apicomplexa protozoans from *Plasmodium* genus, five species infect and cause disease in humans (*Plasmodium falciparum, P. knowlesi, P. malariae, P. ovale, and P. vivax*) (2–4). Their transmission occurs via the bite of the female mosquito of the *Anopheles* genus (5). Approximately 216 million cases of malaria were diagnosed worldwide in 2016, with 445,000 deaths. In Brazil, 175,000 cases were reported in 2017. The Amazon region contributes to nearly 99% of the malaria notifications and *Plasmodium vivax* is responsible for most diagnosed cases (6–8).

The onset of the immune response occurs when the mosquito inoculates the sporozoites in the skin, which may be phagocytosed by dendritic cells and macrophages and presented to the lymphocytes (9). Hemolysis of red blood cells infected with *Plasmodium* is the main pathogenic mechanism observed during infection. Parasites release endotoxins known as hemozoin linked to parasitic DNA that bind to the Toll-like Receptor 9 (TLR-9) and activate the host immune response and the production of several inflammatory mediators against these pathogens (10, 11).

Eicosanoids are lipid mediators of local signaling, derived from arachidonic acid (AA), which participate in homeostatic and inflammatory processes (12, 13). Their production occurs when AA fuels enter three main metabolic pathways: the cyclooxygenases (COX-1 and COX-2) pathway that produces prostaglandins (PGs) and thromboxanes (TXs); the lipoxygenases (5-LO, 12-LO, and 15-LO) pathway producing the leukotrienes (LTs), lipoxins (LPs), hepoxylines and hydroxyethylacetaenoic acids (HETEs); and the cytochrome P450 (CYP450) pathway that produces epoxyleicatriatrienoic acids (EETs), dihydroxyyeatriatrienoic acids (DiHETEs), and HETEs (14). The production of eicosanoids has already been documented in patients infected with *P. falciparum*, and studies suggest that low levels of plasma PGE_2_ are associated with severe disease (15). Moreover, high concentrations of LTB_4_ in mice infected with *Plasmodium berghei* were detrimental to the pathogenesis of cerebral malaria (16).

Previous studies have shown alterations in phospholipase A2 (PLA2) activity, which is involved in the glycerophospholipid metabolism, during *P. vivax* malaria episodes in human and in erythrocytes infected with *P. falciparum* (17). More recently, alterations in glycerophospholipid and glycosphingolipid metabolism was also observed in P. vivax malaria (18). Furthermore, metabolomic data suggest that the host response to *P. vivax* infection might be affected by metabolites involved in the degradation of heme and metabolism of several lipids, including oleic acid and omega-carboxytrinor-leukotriene B4 (19).

The Amazon region comprises the largest and most biodiverse part of the world's tropical forests. The Amazon region comprises the largest and most biodiverse part of the world's tropical forest. It is an endemic area for tropical and infectious diseases with similar clinical spectrum and severity, such fever, headache, and myalgia, favoring the maintenance of the self-medicate culture (20, 21). Because of this, self-medication is a common practice in Brazil and involves the use of medicinal plants and medicines without a prescription. The aim of this research was to describe the profile of lipid mediators and its possible influence on the production of cytokines and chemokines in patients with *P. vivax* malaria (*Pv*-malaria), both in the phase acute and convalescent stage. The data from this research suggest that self-medication influences the production of eicosanoids in patients infected with *P. vivax* and results in a greater interaction between the studied inflammatory mediators.

## Materials and Methods

### Ethics Statement

The study was approved by the Research Ethics Committee at Fundação de Medicina Tropical Dr. Heitor Vieira Dourado (CEP/FMT-HVD process n°.1.358.078/15). All participants read and signed the written informed consent form, in accordance with the Declaration of Helsinki and Resolution 466/12 of the National Health Board for research involving human subjects.

### Healthy Individuals and Patients

Twenty-three patients were included in this study. They were diagnosed with non-severe acute malaria caused by *P. vivax* via a thick blood smear and the diagnosis was confirmed by the 18S rRNA gene using the 7500 Fast qPCR System (Applied Biosystems, Foster, CA, USA) as described previously (22–24) at FMT-HVD, which is located in the city of Manaus, Western Brazilian Amazon. Of these 23, 14 patients reported self-medication with anti-inflammatory drugs prior to diagnosis (SM), and 9 patients stated that they did not take any type of medication until the time of confirmation of the disease (NSM). 12 patients returned to the hospital 28 days (D28) after admission. This period is known as the convalescent stage after-treatment (AT) and is characterized when the individual is not sick, but has not yet fully recovered. This is known as the transition phase between disease and total cure. A total of 12 individuals were selected for the control group, and comprised of healthy donors who lived in the same city and had no previous history of malaria. Exclusion criteria included: patients under 18 years of age, pregnant women, people belonging to the indigenous population, patients with co-infected or already receiving treatment for malaria. All study participants underwent serological screening recommended by all Brazilian blood banks and this was carried out at the Fundação Hospitalar de Hematologia e Hemoterapia do Amazonas (HEMOAM) serology laboratory for the purpose of exclusion. The clinical and demographic data were acquired using a standardized questionnaire.

### Collection, Processing and Transport of Biological Samples

Whole blood samples from healthy subjects and patients were collected in tubes containing EDTA (BD Vacutainer^®^ EDTA K2 Franklin Lakes, New Jersey, USA) and tubes containing Sodium Heparin (Labor Import^®^ São Paulo, Brazil). Samples were then stored in isolated boxes for biological material and transported to the laboratory, where samples collected with EDTA were centrifuged in a refrigerated centrifuge (5702R, Eppendorf, Hamburg, DEU) for 5 min at 1,900 g. The obtained plasma was separated and placed in cryotubes and stored in a freezer at −80°C for further quantification of cytokines and chemokines.

Tubes containing sodium heparin were stimulated with 20 μM Thapsigargine (SIGMA-ALDRICH Life Science) for 20 min in a water bath at 37°C under agitation. After this period, the reaction was blocked in an ice bath, and the stimulated whole blood was centrifuged at 400 g for 20 min at 4°C. The acquired plasma (300 μL) was aliquoted into cryotubes and immediately frozen at −80°C (25). These frozen aliquots were transported on dry ice to the Laboratory of Inflammation and Immunology of Parasitoses (LIIP), located at the School of Pharmaceutical Sciences of Ribeirão Preto (FCFRP-USP) and later submitted to high performance liquid chromatography-mass spectrometry (HPLC-MS/MS).

All samples were stored and discarded after the experiments, obeying the norms of Good Clinical and Laboratorial Practices (GCLP).

### Preparation of Plasma Samples for HPLC-MS/MS

The extraction of the eicosanoids in the plasma samples was performed by the solid phase extraction (SPE) method. In summary, the 12-epi-LTB_4_-d4 analyte acting as internal standard (PI) was added to the plasma samples, then these samples were denatured overnight with 1.5 mL of an ice-cold methanol/acetonitrile (1–1, v-v). The denatured proteins were removed by centrifugation at 400 g for 20 min at 4°C and the supernatants obtained (~1.2 ml) were diluted in 14 ml of ultra-pure water to decrease the methanol/acetonitrile content to 15% between organic phase and aqueous phase. Purification of the eicosanoids was performed by a Waters^®^ Extraction Manifold and Sep-Pak C18 cartridges, 500 mg sorbent, 2.8 ml, HyperSep™ (Thermo Scientific, USA). Samples were transferred to pre-activated cartridges with 2 mL of methanol and 2 mL of 0.1% (v-v) water/acetic acid solution. After elution of the plasma samples, 2 mL of water −0.1% acetic acid (v-v) was added to the cartridge to elute hydrophilic polar compounds and, finally, the eicosanoids adhered on C18 silica were eluted with 2 mL of methanol/0.1% acetic acid (v-v) solution. The solvent was removed under reduced pressure at speedvac, and 100 μL of methanol was added to the extract obtained and transferred to HPLC-MS/MS (25).

### Qualitative and Quantitative Analysis of Eicosanoids by HPLC-MS/MS

For this type of analysis, an Acquity™ UPLC (Waters^®^) system was used consisting of a quaternary pump and automatic injector coupled to the Xevo TQ-S mass spectrometer with ESI ionization source equipped with Orthogonal Z-spray (Waters^®^). The chromatographic analyses were performed on an Ascentis EXPRESS C18 column (100 × 4.6 mm, 2.7 μm) using a flow rate of 0.6 mL/min at 25°C. Elution was done using a binary gradient system consisting of water: acetonitrile: acetic acid (69.98–30-0.02, v-v-v) (Step A) and acetonitrile: isopropanol (70–30. v-v) (Stage B) with 0% B from 0 to 2 min, increasing to 15% B at 2 min, 20% B at 5 min, 35% B at 8 min, 40% B at 11 min, 100% B at 15 min and remained in this proportion up to 19 min. From this time up to 30 min, the gradient returned to the initial ratio for column conditioning. The ionization source operated in negative mode and by Multiple Reaction Monitoring (MRM). To optimize the MRM conditions, a standard solution of each eicosanoid at 100 ng/mL diluted in methanol/water/ammonium acetate (71-30-0.1, v-v-v) was infused into the MS at 10 μL/min and fragmented by dissociation induced by collision with argon in order to obtain the spectrum of the ion product of each analyte. After selection of the MRM transitions, the cone and collision energies were standardized through the direct infusion of the standards to obtain adequate sensitivity. The linearity was evaluated through a calibration curve, in triplicate, of plasma samples enriched with standard solutions of LTB_4_, 6-trans-LTB_4_, LTD_4_, 11-trans-LTD_4_, LTE_4_, PGD_2_, PGE_2_, 20-OH- PGE_2_, 15-keto-PGE_2_, 15-deoxy-Δ12,14-PGJ_2_, PGJ_2_,5-HETE, 12-HETE, 15-HETE, 20-HETE, (+) 11,12-DHET, 15-DHET, 5-Oxo-ETE, 11,12-EET, 5 (S), 6 (R) -DiHETE, LXA_4_, and TXB_2_ (Cayman Chemical) and extracted by the method described in the above item, of 5.0; 7.5; 15.0; 30.0; 50.0, and 75.0 ng/ml. A linearity curve was also constructed for LTB_4_, 5-HETE, 12-HETE, and TXB_2_ (Cayman Chemical) contemplating the following concentrations: 75.0; 150.0; 300.0; 450.0; 800.0, and 1000.0 ng/ml. The calibration graph was constructed by plotting the plasma concentration values of each eicosanoid on the abscissa axis and the ratio between the area of the peaks obtained for each mediator and the area of the PI on the axis of the ordinates. Data analysis was done via linear regression using the least squares method, where the area/concentration relation is expressed by the equation of the line (y = ax + b), where is the angular coefficient and b is the linear coefficient of the line. The correlation coefficient (r) was also calculated from the experimental points, which is the parameter that estimates the linearity of the calibration curve. Masslynx 4.1 software (Micromass, Manchester, UK) was used for data acquisition and processing.

### Quantification of Plasma Concentrations of Circulating Cytokines and Chemokines

The concentrations of chemokines (CXCL-8, CCL-2, CCL-5, CXCL-9, and CXCL-10) and circulating cytokines (IL-2, IL-4, IL-6, IL-10. TNF, IFN-γ, and IL-17A) through the CBA (Cytometric Bead Array) BD™ Human Chemokine Kit and CBA (Cytometric Bead Array) BD™ Human Th1, Th2, Th17 Cytokine kit, respectively. IL-1β and IL-5 cytokines were quantified by ELISA (BD OptEIA II Human Kit, BD Biosciences Pharmingen, USA), following the manufacturer's instructions.

### Statistical Analyses

Clinical, epidemiological, laboratory, lipid mediator, chemokine, and cytokine concentrations were tabulated and stored using Microsoft Excel^®^ Software (2010 version for Windows). The descriptive analyses were presented in tables and graphs of frequency distribution, elaborated in GraphPad Prism 5.0 software (San Diego, CA, USA), taking into account that the data have a non-parametric distribution. The comparative analyses between the groups were performed using the Mann-Whitney (NSM vs. HD, SM vs. HD, NSM vs. SM) and Wilcoxon test (NSM and SM vs. AT), with significant values being set at *p* < 0.05. From analysis of the Spearman correlation between the biomarkers, the networks analysis and demonstration of the complex interactions between chemokine, cytokines, and lipid mediators were performed in all groups of this study using Cytoscape software 3.0.4 (Cytoscape Consortium San Diego, CA, USA). The thickness of the lines was adjusted to portray the strength and whether the type of correlations was positive or negative.

## Results

### Demographic, Parasitological, and Baseline Clinical Characteristics of the Study Population

All subjects in this study reside in an area of intense malaria transmission. Although they are in contact with *P. vivax* malaria (*Pv*-malaria) transmission vectors, 28% of the recruited individuals were primo-infected. The median age between the control and *Pv*-malaria groups was similar (33 and 39 years [*p* = 0.144], respectively), with male subjects predominating (58 and 74% [*p* = 0.450], respectively). The average parasite load was 127,574 parasites/μL, and was similar among patients who did not Self-Medicate (NSM) and those who Self-Medicated (SM) (*p* = 0.361). Furthermore, median of the number of infections was 2 episodes, without any difference between the *Pv*-malaria subgroups (*p* = 0.303). Table 1 summarizes the demographic, parasitological, and clinical characteristics.

**Table 1:** Demographic, parasitological, and baseline clinical characteristics of Control Group (CG) and *Pv*-malaria patients that did Not Self-Medicate (NSM) and Self-Medicated (SM).

**Variables**	**CG (*n* = 12)**	***Plasmodium vivax*** **malaria**		
		**All (*n* = 23)**	**NSM (*n* = 9)**	**SM (*n* = 14)**
Age (years, median [IQR])	33 [27–41]	39 [30–54]	39 [35–52]	43 [23–54]
Gender (male/female)	7/5	17/6	9/0	8/6
Parasitemia (number of copies/μL, median [IQR])	−	127,574 [28,242–637,392]	236,429 [109,717–604,622]	68,602 [19,896–664,631]
First infection (yes/no)	−	5/18	1/8	4/10
Number of infections (median [IQR])	−	2 [2–9]	4 [2–9]	2 [1–6]
Headache (yes/no)	−	21/2	8/1	13/1
Fever (yes/no)	−	12/11	6/3	6/8
Myalgia (yes/no)	−	12/11	5/4	7/7
Chills (yes/no)	−	6/17	2/7	4/10

### Hematological and Biochemical Parameters of *Pv*-Malaria Patients That Did Not Self-Medicate (NSM) and Self-Medicated (SM)

Table 2 summarizes the hematological and biochemical parameters which were evaluated in the subgroups of *Pv*-malaria and control group (CG). No significant differences were observed among patients than did not Self-Medicate (NSM) and Self-Medicated (SM), except for differences in hematocrit and uric acid concentrations. Furthermore, the CG had significantly higher/lower concentrations and numbers of hematocrit, red cell distribution width, white blood cells, eosinophil, lymphocyte, monocyte, mean platelet volume, platelet, total cholesterol, HDL, LDL, uric acid and total, direct and indirect bilirubin when compared to NSM and SM groups.

**Table 2:** Hematological and Biochemical parameters of Control Group (CG) and *Pv*-malaria patients that did not Self-Medicate (NSM) and Self-Medicated (SM).

**Variables**	**CG_**(a)**_ (*n* = 12)**	***Plasmodium vivax*** **malaria**		*p*-value^*^
		**NSM_**(b)**_ (*n* = 9)**	**SM_**(c)**_ (*n* = 14)**	
RBC^#^ (unit/mm^6^, median [IQR])	4.9 [4.5–5.1]	4.9 [4.6–5.2]	4.5 [4.2–4.9]	0.107
HB (g/dL, median [IQR])	14.0 [13.0–15.6]	14.4 [13.3–15.4]	12.9 [12.6–14.5]	0.075
HT (%, median [IQR])	41.3 [38.7–45.7]	41.3 [39.3–44.4]	38.4 [36.1–41.9]	**0.047**_**ab, bc**_
MCV (fL, median [IQR])	86.1 [82.9-90.0]	85.7 [84.7–90.0]	85.8 [84.1–87.3]	0.921
MCH (pg, median [IQR])	29.4 [28.7–30.6]	30.2 [28.6–31.3]	29.7 [28.8–30.3]	0.871
MCHC (g/dL, median [IQR])	34.1 [33.3–34.9]	34.6 [34.1–35.0]	34.7 [34.3–34.8]	0.373
RDW (%, median [IQR])	13.4 [12.9–13.5]	14.0 [13.6–14.4]	13.6 [13.1–14.4]	**0.048**_**ab**_
WBC (unit/mm^6^, median [IQR])	7.2 [6.5–8.3]	5.7 [4.6–7.1]	5.0 [4.2–6.6]	**0.020**_**ac**_
Neu (unit/mm^6^, median [IQR])	4.2 [3.3–4.8]	3.7 [2.8–5.5]	3.3 [2.3–4.7]	0.454
Eos (unit/mm^6^, median [IQR])	0.3 [0.2–0.3]	0.1 [0.02–0.2]	0.1 [0.07–0.1]	**0.004**_**ab, ac**_
Lym (unit/mm^6^, median [IQR])	2.4 [1.9–2.9]	1.0 [7.3–1.5]	0.9 [0.8–1.8]	**0.001**_**ab, ac**_
Mon (unit/mm^6^, median [IQR])	0.5 [0.4–0.5]	0.4 [0.3–0.6]	0.3 [0.2–0.4]	**0.031**_**ac**_
PLT (unit/mm^6^, median [IQR])	260 [207–329]	93.0 [54.0–121]	106 [87.8–179]	**<0.0001**_**ab, ac**_
MPV (fL, median [IQR])	7.6 [7.1–8.4]	9.2 [7.7–10.1]	7.3 [7.0–8.6]	0.083
Total lipids (mg/dL, median [IQR])	635 [550–759]	577 [466–714]	549 [522–628]	0.172
Triglycerides (mg/dL, median [IQR])	173 [81.8–308]	109 [85.0–271]	153 [101–228]	0.916
Total cholesterol (mg/dL, median [IQR])	177 [145–207]	120 [101–193]	120 [93.5–131]	**0.006**_**ac**_
HDL (mg/dL, median [IQR])	47.0 [39.5–52.8]	27.0 [7.0–38.5]	14.5 [8.8–36.5]	**0.0003**_**ab, ac**_
LDL (mg/dL, median [IQR])	102 [78.3–120]	76.0 [21.5–118]	63.5 [48.8–69.0]	**0.008**_**ac**_
VLDL (mg/dL, median [IQR])	35.5 [22.0–61.8]	21.0 [8.5–44.5]	30.5 [20.0–45.3]	0.326
Glucose (mg/dL, median [IQR])	109 [92.4–135]	94.0 [85.0–120.5]	93.5 [63.0–109]	0.234
Uric acid (mg/dL, median [IQR])	4.3 [3.6–5.9]	5.3 [4.6–5.7]	3.4 [0.0–4.4]	**0.035**_**bc**_
Creatinine (mg/dL, median [IQR])	0.9 [0.8–1.0]	1.1 [0.9–1.1]	0.9 [0.7–1.0]	0.117
Urea (mg/dL, median [IQR])	30.2 [24.4–33.8]	33.2 [26.7–40.3]	28.5 [20.5–37.3]	0.511
GGT (U/I, median [IQR])	28.4 [22.2–65.2]	66.6 [23.3–146]	47.0 [36.3–160]	0.093
ALP (U/I, median [IQR])	77.8 [71.1–98.7]	84.1 60.2–93.0]	85.5 [78.0–113]	0.322
AST (U/I, median [IQR])	32.9 [30.0–44.5]	39.8 [23.8–47.6]	29.5 [22.5–38.9]	0.333
ALT (U/I, median [IQR])	26.9 [22.6–39.3]	35.0 [18.0–44.1]	25.5 [19.0–39.9]	0.840
Total bilirubin (mg/dL, median [IQR])	0.3 [0.2–0.5]	1.1 [0.8–1.8]	1.5 [0.4–1.8]	**0.018**_**ac**_
Direct bilirubin (mg/dL, median [IQR])	0.1 [0.1–0.2]	0.4 [0.2–0.7]	0.6 [0.2–0.7]	**0.015**_**ac**_
Indirect bilirubin (mg/dL, median [IQR])	0.2 [0.1–0.3]	0.8 [0.5–1.1]	0.9 [0.2–1.1]	**0.026**_**ab, ac**_

* *Statistical analyses were performed by the Kruskal-Wallis test, followed by Dunn's test in order to compare pairs; ^#^RBC, Red blood cells; HB, Hemoglobin; HT, Hematocrit; MCV, Mean corpuscular volume; MCH, Mean corpuscular hemoglobin; MCHC, Mean corpuscular hemoglobin Concentration; RDW, Red cell distribution width; WBC, White blood cells; Neu, Neutrophil; Eos, Eosinophil; Lym, Lymphocyte; Mon, Monocyte; PLT, Platelet; MPV, Mean platelet volume; HDL, High Density Lipoprotein; LDL, Low Density Lipoprotein; VLDL, Very Low Density Lipoprotein; GGT, Gamma Glutamyl Transferase; ALP, Alkaline Phosphatase; AST, Aspartate Aminotransferase and ALT, Alanine Aminotransferase*.

### Plasmatic Profile of Eicosanoids in *P. vivax* Malaria

**Figure 1:**
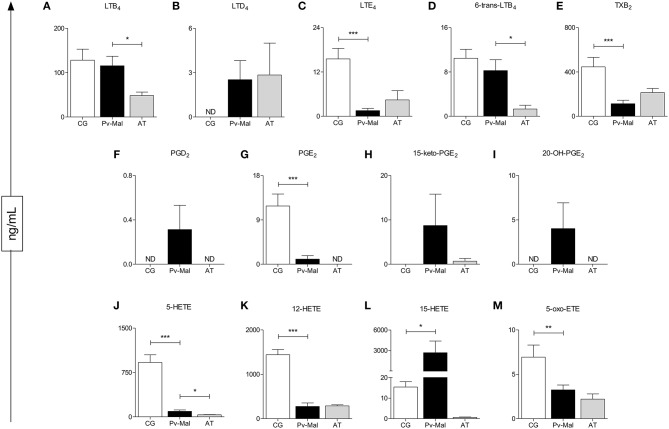
Identification and quantification of eicosanoids after stimulation with Thapsigargine, from control group (white bar), *Pv*-malaria patients (black bar) and patients who returned 28 days after treatment, understood as the period of convalescence (gray bar). ^*^p < 0.05, ^**^p < 0.01, ^***^p < 0.0001 between groups studied. ND = Concentration not detected. The quantifications of these lipid mediators were measured using high performance liquid chromatography-mass spectrometry (HPLC-MS).

Analysis of the plasmatic profile of eicosanoids after whole blood stimulation indicated that *P. vivax* infection may influence the serum concentrations of these biomolecules (Figure 1). The evaluation of these lipid mediators demonstrated a significant decrease of LTE_4_, PGE_2_, 5-HETE, 12-HETE, 5-Oxo-ETE, and TXB_2_ (Figures 1C,F,I,J,L,M) in patients with *Pv*-malaria. In addition, increased levels of LTD_4_, PGD_2_, 15-keto-PGE_2_, 20-OH-PGE_2_, and 15-HETE were observed in these individuals (Figures 1B,E,G,H,K). We observed significantly lower concentrations of LTB_4_, 6-trans-LTB_4_, and 5-HETE when comparing the prior (*Pv*-malaria) and convalescent stage after-treatment (AT) patients (Figures 1A,D,I), with the increase of 12-HETE and TXB_2_ (Figures 1J,M).

### Influence of Self-Medication on the Circulating Concentration of Eicosanoids in *Pv*-Malaria

**Figure 2:**
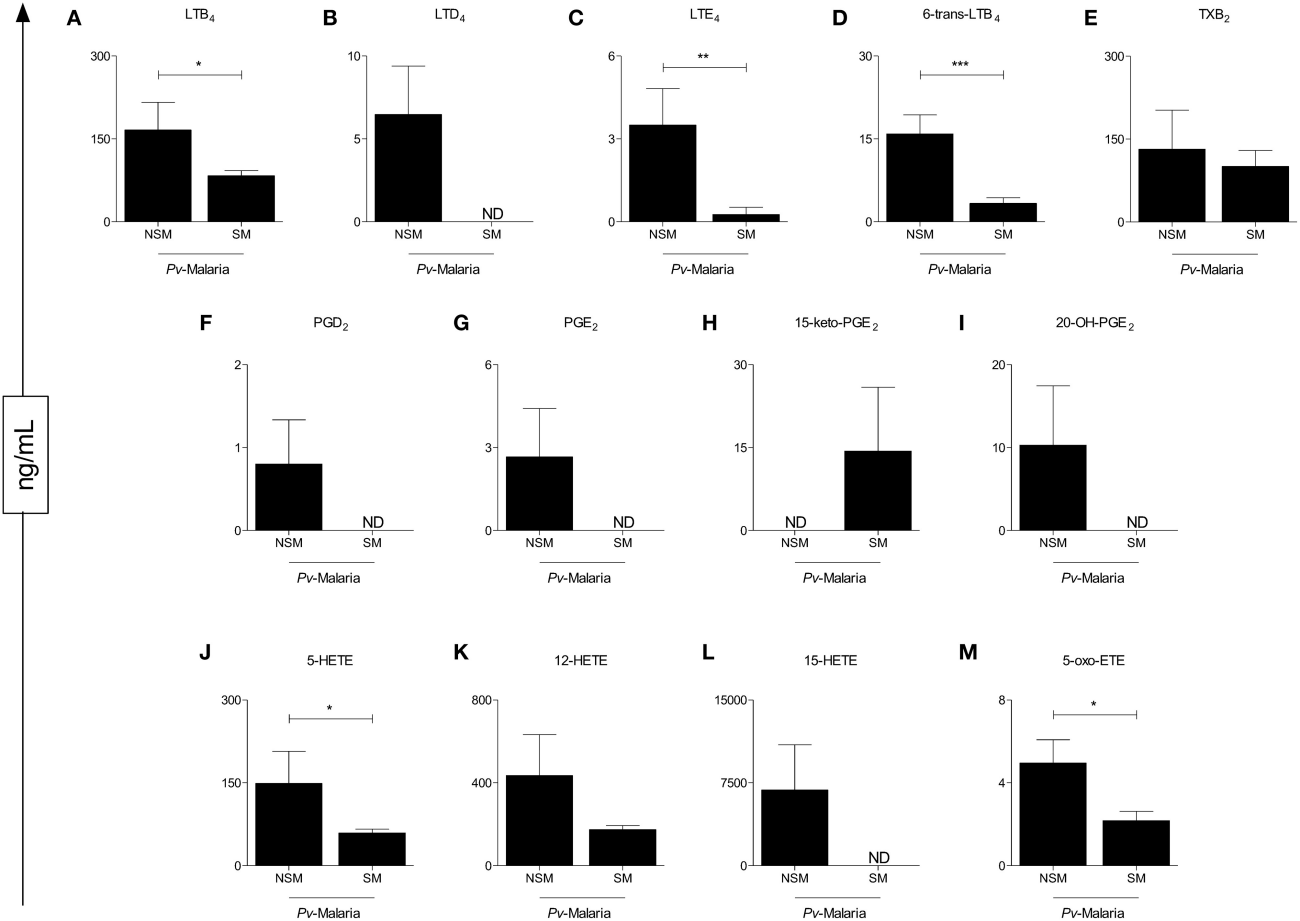
Analysis of the eicosanoid concentrations of *Pv*-malaria patients at the time of diagnosis who did not Self-Medicate (NSM) and Self-Medicated (SM). ^*^p < 0.05, ^**^p < 0.01, ^***^p < 0.0001 between groups studied. ND = Concentration not detected. The quantifications of these lipid mediators were measured using high performance liquid chromatography-mass spectrometry (HPLC-MS).

To understand the influence of self-medication on eicosanoid levels in *Pv*-malaria, we separated the patients into two groups: No Self-Medication (NSM) and Self-Medication (SM) *Pv*-malaria patients (Figure 2). A significant reduction of LTB_4_, LTE_4_, 6-trans-LTB_4_, 5-HETE, and 5-Oxo-ETE (Figures 2A,C,D,I,L) was observed in SM *Pv*-malaria patients. In addition, the eicosanoids LTD_4_, PGD_2_, PGE_2_, 20-OH-PGE_2_, and 15-HETE (Figures 2B,E,F,H,K) were not detected in these individuals. 12-HETE and TXB_2_ were not significantly different between groups (Figures 2J,M).

### Plasmatic Profile of Chemokines and Cytokines in *Pv*-Malaria and the Influence of Self-Medication in These Patients

**Figure 3:**
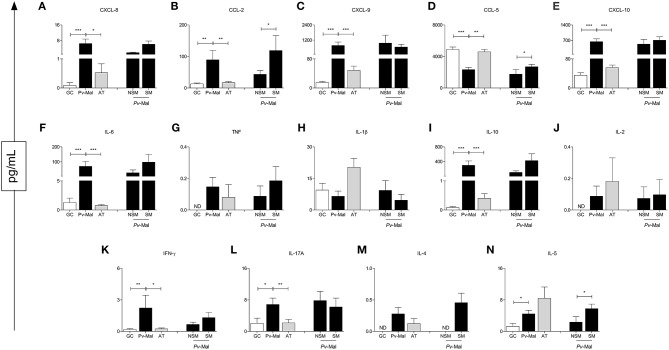
Concentration of chemokines and cytokines in control group (white bar), *Pv*-malaria patients (black bar) and patients who returned 28 days after treatment, understood as the period of convalescence (gray bar). In addition, analysis of the chemokines and cytokines concentrations of *Pv*-malaria patients at the time of diagnosis who did not Self-Medicate (NSM) and Self-Medicated (SM). ^*^p < 0.05, ^**^p < 0.01, ^***^p < 0.0001 between groups studied. ND = Concentration not detected. The quantifications of these chemokines and cytokines were measured using Cytometric Bead Array (CBA) and ELISA.

Our data demonstrated that patients with *P. vivax* malaria presented chemokine and cytokine storms during infection (Figure 3). A significant increase of CXCL-8, CCL-2, CXCL-9, CXCL-10. IL-6, IL-10. IFN-γ, IL-17A, and IL-5 (Figures 3A–C,E,F,I,K,L,N) were observed in these patients. Although this phenomenon is observed at the end of the convalescence stage after treatment, the circulating levels reestablish themselves and reach the concentrations of the control group (Figure 3). To further characterize these immunological markers, chemokine levels and plasma cytokines were analyzed in NSM and SM patients. It is observed that the use of the drugs positively influenced the production of CCL-2, CCL-5, and IL-5 (Figures 3B,D,N).

### *Plasmodium vivax* Malaria and Self-Medication (SM) Change the Interaction of Eicosanoids, Chemokines, and Cytokines During Infection

**Figure 4:**
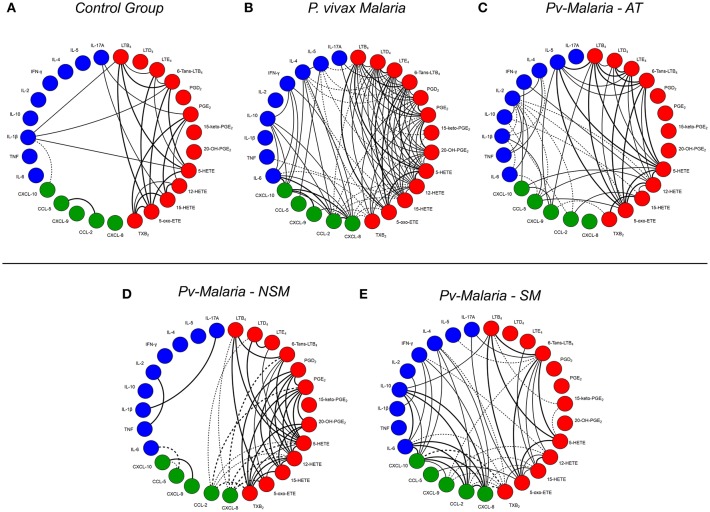
A network of interaction between eicosanoids, chemokines and cytokines in control group, *Pv*-malaria patients, individuals who returned 28 days after treatment, understood as the period of convalescence and *Pv*-malaria patients at the time of diagnosis who did not Self-Medication (NSM) and Self-Medicated (SM). Each connecting line is a significant correlation between a pair of markers. Continuous lines represent positive correlations, while dashed lines represent negative correlations (*p* < 0.05). The degree of significance is represented by the thickness of the line. Correlations were obtained through the Spearman test; the value of r and p were used to classify the connections as weak (*r* ≤ 0.35, *p* < 0.05), moderate (*r* = 0.36–0.67, *p* < 0.01) or strong (*r* ≥ 0.68, *p* < 0.0001). The absence of the line indicates the non-existence of the relationship.

To evaluate the relationship between levels of eicosanoids, chemokines, and cytokines during *P. vivax* infection, a series of correlation analyses were performed (Figure 4). When comparing the analyzed interactions between molecules in the *Pv*-malaria and control group, there was an intense increase in relations, especially the chemokine (CXCL-8, CCL-2 and CXCL-10) and cytokines (IL-6, IL- 10. IFN-γ, IL-4, and IL-5) (Figure 4B). In addition, the number of interactions between the analyzed eicosanoids also increased during *P. vivax* infection, but this was not observed in the control group (Figure 4A). This phenomenon remains even in the convalescent stage after-treatment (Figure 4C). Finally, we performed the analysis of the correlation indices by categorizing patients with *Pv*-malaria in two different groups (NSM and SM) and observed that the interactions were influenced in individuals who self-medicated. Decreased eicosanoid ratios and increased correlations with chemokines and cytokines were noted (Figure 4E).

## Discussion

In this study, we characterize the eicosanoid profile of patients with *Pv*-malaria during the acute phase and convalescent stage after-treatment. Moreover, we report the influence of self-medication on this profile. These findings are particularly relevant in order to better understand the interaction between inflammatory and lipid mediators during the cure of malaria-*vivax* infection. We demonstrate that *Pv*-malaria infection significantly affects the levels of metabolites derived from LO or COX pathways. Levels of these lipid mediators are tightly correlated with plasma levels of cytokines and chemokines that are related to clinical characteristics of the patients. Moreover, our data suggest that self-medication (SM) contributes to changes in the correlations between eicosanoids, chemokines, and cytokines during infection, which may aid in parasitic clearance and/or symptom alleviation. To our knowledge, this is the first study to comprehensively profile eicosanoid production and their associations with cytokines and chemokines during infections with *P. vivax*.

*Plasmodium vivax* infection induces inflammatory and regulatory processes that are designed to contain the parasite multiplication, while maintaining the integrity of tissues and organs (26–28). Although most patients with *P. vivax* malaria exhibit mild clinical symptoms, severe cases are reported and have been associated with elevated levels of TNF-α, IFN-γ and low levels of PGs, TXBs, and IL-10 (29–33). Our data further extend these findings by demonstrating reduced a production of PGE_2_ even during mild cases of *P. vivax* infection. PGE_2_ suppresses type I interferon responses, while *P. vivax* infection promotes robust transcriptional interferon signatures, which are associated with linoleate metabolism (34) and a pathway that influences arachidonic acid biosynthesis. Furthermore, TXB_2_, an inactive product derived from TXA_2_, was also reduced during *P. vivax* infection and might reflect the degree of thrombocytopenia in these patients (35). This is further supported because treatment recovered platelet counts, while only levels of TXB_2_ increased during convalescence.

Treatment down-regulated the lypoxygenase pathways, reducing the production of LTB_4_, 6-trans-LTB_4_, 5-HETE, and 12-HETE. Except for the last lipid mediator which is exclusively produced by action of 12-LO, since formation of the other eicosanoids involves reactions catalyzed by 5-LO. All these metabolites were negatively correlated with the Th2 cytokine profiles, IL-4 and/or IL-5. After-treatment changed these correlations, whereby IL-5 became positively correlated with LTB_4_, 6-trans-LTB_4_ and 5-HETE. IL-5 signaling has been shown to promote 5-LO activating protein (FLAP) expression and 5-LO activation and nucleus translocation (36). Self-medication reduced the production of these mediators, whose 6-trans-LTB_4_ was also negatively correlated with IL-5. These data highlight an underlying association between Th2 cytokine profiles and LOX products that can be relevant to the treatment of *P. vivax* malaria. A recent study has demonstrated that a major product of LTB_4_ catabolism by neutrophils, omega-carboxy-tinor-LTB_4_, is significantly associated with *P. vivax* parasite burden, which suggests that the production of eicosanoids might also be affected by clinical parameters such as the degree of parasitemia (19).

We observed little association between the production of eicosanoids and circulating cytokines in the control group, but, in particular, IL-1β was positively correlated with levels of LTB_4_, 6-trans-LTB_4_, and 5-HETE. This is interesting because depending on the context, LTB_4_ exerts a regulatory role over IL-1β production (37, 38). This suggests that 5-LO activity might also be important in order to limit IL-1β release during homeostatic state, of which levels increased during convalescence, when 5-LO metabolites were also reduced. After *P. vivax* infection, there were no significant correlations with IL-1β, which did not recover after treatment. However, further sub-classification of patients demonstrated that IL-1β positively correlated with IL-17 during infection of NSM patients but not SM patients, although both groups exhibited similar levels of these cytokines. This is consistent with the role of IL-1β for the differentiation and polarization of naïve T lymphocytes into Th17 cells (39), whereas lack of correlation after self-medication suggests a disruption in this process. Levels of IFN-γ and the related chemokine, CXCL-10. increased after *P. vivax* infection. IFN-γ did not correlate with lipid mediators during acute infection for all patients, but self-medication changed this profile, showed inverse correlations between IFN-γ and 12-HETE in the interaction network between immunological molecules and lipid mediators (Figure 4). During convalescence, significant correlations were also observed between IFN-γ, CXCL-10, and 12-HETE, suggesting a potential cross-talk between these mediators.

The local population is very well-informed about malaria and knows the need for diagnosing malaria at the onset of the disease. However, the study has limitations since eicosanoids, chemokines, and cytokines were not evaluated at the onset of infection and/or symptoms. Furthermore, the small sample size does not allow intra-comparison of the eicosanoids, chemokines, and cytokines with clinical manifestations of *Pv*-malaria, including in asymptomatic and severe malaria.

## Conclusion

In summary, *P. vivax* infection triggers production of immune and lipid mediators, generally influencing the increase of these molecules in the acute stage of the disease. Our data also suggest that after antimalarial treatment, the serum levels of these molecules are reestablished. Furthermore, self-medicated individuals had a significant impact on the production of lipid mediators, chemokines and cytokines, altering the interaction dynamics between these molecules in *Pv*-malaria infection. Finally, we believe that self-medication deserves more attention during disease follow-up, since most of the drugs consumed are exempt from prescription, but aren't exempt from risk.

## Data Availability

The raw data supporting the conclusions of this manuscript will be made available by the authors, without undue reservation, to any qualified researcher.

## Ethics Statement

All protocols and consent forms were approved by the Research Ethics Committee at the FMT-HVD (CEP/FMT-HVD process #1.358.078/2015). Patients were treated according to recommendations of the Brazilian Health Ministry.

## Author Contributions

PA-F, AT, AC, LG, LF, and AM designed and performed the experiments, analyzed data, and wrote the manuscript. PA-F and AC analyzed data. AFGM, TC, FZ, LG, and LM performed the experiments. CS analyzed the data and revised the manuscript. PA-F, WM, ML, LF, and AM conceived and supervised the project, designed the experiments, interpreted the data, wrote and revised the manuscript. All authors read and approved the final manuscript.

### Conflict of Interest Statement

The authors declare that the research was conducted in the absence of any commercial or financial relationships that could be construed as a potential conflict of interest.
